# The Goodwin Model: Behind the Hill Function

**DOI:** 10.1371/journal.pone.0069573

**Published:** 2013-08-01

**Authors:** Didier Gonze, Wassim Abou-Jaoudé

**Affiliations:** 1 Faculté des Sciences, Université Libre de Bruxelles, Bruxelles, Belgium; 2 Institut de Biologie, Ecole Normale Supérieure, Paris, France; University of Sheffield, United Kingdom

## Abstract

The Goodwin model is a 3-variable model demonstrating the emergence of oscillations in a delayed negative feedback-based system at the molecular level. This prototypical model and its variants have been commonly used to model circadian and other genetic oscillators in biology. The only source of non-linearity in this model is a Hill function, characterizing the repression process. It was mathematically shown that to obtain limit-cycle oscillations, the Hill coefficient must be larger than 8, a value often considered unrealistic. It is indeed difficult to explain such a high coefficient with simple cooperative dynamics. We present here molecular models of the standard Goodwin model, based on single or multisite phosphorylation/dephosphorylation processes of a transcription factor, which have been previously shown to generate switch-like responses. We show that when the phosphorylation/dephosphorylation processes are fast enough, the limit-cycle obtained with a multisite phosphorylation-based mechanism is in very good quantitative agreement with the oscillations observed in the Goodwin model. Conditions in which the detailed mechanism is well approximated by the Goodwin model are given. A variant of the Goodwin model which displays sharp thresholds and relaxation oscillations is also explained by a double phosphorylation/dephosphorylation-based mechanism through a bistable behavior. These results not only provide rational support for the Goodwin model but also highlight the crucial role of the speed of post-translational processes, whose response curve are usually established at a steady state, in biochemical oscillators.

## Introduction

Biological rhythms are generated, at the cellular level, by complex gene-protein interaction networks. Their molecular mechanism is characterized by regulatory feedback loops and non-linear dynamics [1]. Non-linearity typically arises from Michaelis-Menten enzyme kinetics and cooperative processes. Sigmoidal responses and sharp thresholds may result from the cooperative binding of a substrate molecule to an enzyme or from post-translational modifications, such as multisite phosphorylation [2]–[4]. In some conditions, even a single, reversible phosphorylation can produce a step-like response [5]. Cooperative binding of transcription factors to various sites of a gene promoter or the formation of transcription factor multimers can account for non-linearity in gene transcription [6].

Mathematical models for biological oscillators accomodate these non-linear kinetics either explicitly (using detailed reactional schemes built on mass action laws), or phenomenologically (through Michaelis-Menten or Hill functions). The latter is often privilegiated because detailed molecular mechanisms are generally not known and because it greatly simplifies the models. Indeed, relying - often implicitely - on quasi-steady state assumptions, phenomenological models allow to relinquish fast-varying variables, thereby reducing the number of variables. Since the numerical integration of detailed systems involving a mixture of fast and slow processes may rapidely become prohibitive (CPU-consuming, loss of numerical accuracy), simplifications are highly desired. If appropriate assumptions are done, the reduced version of a model is expected to yield consistent results with its detailed version [7].

In 1965, Goodwin [8] proposed a phenomenological, 3-variable model to show the possibility of oscillations in a simple delayed negative feedback loop model. Synthesis and degradation rates are linear, except the repression which takes the form of a sigmoidal Hill curve. Griffith [9] demonstrated that limit-cycle oscillations can be obtained only if the Hill coefficient 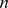 is larger than 8. Since then, several theoretical works investigated the dynamical properties of this model [10]–[13].

The Goodwin model is a prototypical biological oscillator. It was initially presented as an hypothetical genetic oscillator, in which a protein represses the transcription of its own gene via an inhibitor. This model was subsequently applied in the context of circadian clocks [14], [15] and somitogenesis [16]. Many models for circadian clocks are closely related to the Goodwin model [17]–[19]. In particular, one variant of the Goodwin model, in which the Hill function is replaced by an arbitrary 2-threshold “reset” function [20], [21], has been used to reproduce phase response curves and to study temperature compensation in circadian systems.

The Goodwin model, however, is often criticized because of the “unrealistic” large value of the Hill coefficient 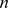. Uses and misuses of the Hill function is regularly revisited [22], [23]. In enzyme kinetics, this coefficient is usually interpreted as the number of ligand molecules that an enzyme or a receptor can bind (in fact the number of binding sites can be shown to be the upper limit of 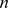 [22], [24], [25]). At the transcriptional level, Hill function can be explained by the formation of repressor protein complexes or the cooperative binding of the repressor to the gene promoter [6]. All these processes rarely yield Hill coefficients higher than 3 or 4.

On the other hand, several recent papers describe molecular mechanisms that can account for sharp thresholds in protein activation kinetics. Mechanisms based on protein sequestration or multisite phosphorylation have been shown to produce sigmoidal responses [2], [3], [26]–[29]. Cascades of post-translational modifications constitute another efficient mean to convert gradual inputs into ultrasensitive responses, equivalent to cooperative enzymes with large Hill coefficients [30]. Such a mechanism likely operates in the MAPK signalling pathway [30], whose switch-like response appears crucial for the cell fate in *Xenopus* oocytes [31].

The goal of the present work is to propose a molecular mechanism of the standard and phase resetting Goodwin model that could explain the switch-like behavior of the repression process while keeping the oscillatory properties of the Goodwin models. We here focus on three mechanisms based on phosphorylation/dephosphorylation processes which have been shown to generate switch-like responses: a multisite phosphorylation/dephosphorylation mechanism which produces Hill-like response, a single phosphorylation/dephosphorylation mechanism which produces zero-order ultrasensitivity and a double phosphorylation/dephosphorylation mechanism giving rise to bistability. When the phosphorylation/dephosphorylation processes are fast enough, we show that the first two mechanisms can provide a molecular validation of the standard Goodwin model, whereas the third one can explain the phase resetting Goodwin model. In particular, limit-cycle generated with the multisite phosphorylation mechanism is in very good quantitative agreement with the oscillations observed in the Goodwin model. The conditions on the parameter values under which a good approximation of the detailed and compact models can be achieved are also discussed. More generally, our work provides a rational support for the use of Hill kinetics in biochemical models and highlights the importance of the speed of the underlying mechanisms explaining threshold and bistable processes in the setting of oscillatory behavior.

## Results

### Limit-cycle Oscillations in the Goodwin Model

**Figure 1:**
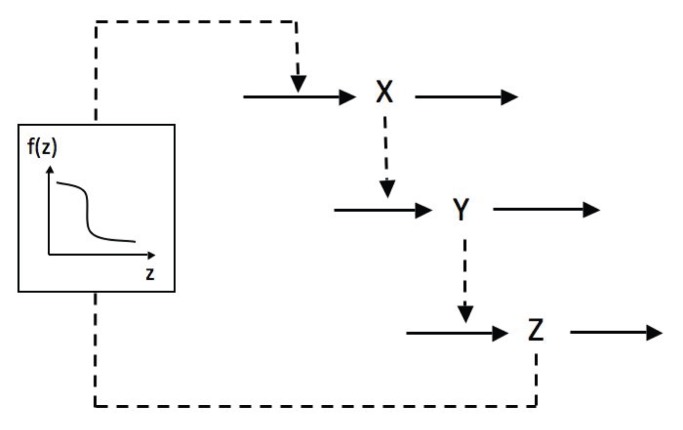
Scheme of the Goodwin model. In the original version of the model, the negative feedback exerted by Z on the synthesis of X is described by a non-linear Hill function.



The Goodwin model [8], [9] is a simple delayed negative feedback loop model (Fig. 1). Its dynamics is governed by 3 ordinary differential equations:

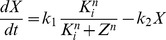
(1)

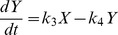
(2)

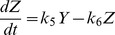
(3)


The variables 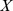, 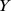 and 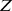 can be interpreted as the concentration of a given gene mRNA, the corresponding protein, and a transcriptional inhibitor, respectively. The feedback loop is achieved by the repression exerted by the inhibitor to the mRNA synthesis and is described by a Hill function:
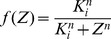
(4)


In 1968, Griffith [9] demonstrated that limit-cycle oscillations can be obtained only if the Hill coefficient 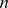 is larger than 8. For 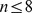, the model displays damped oscillations.

**Figure 2:**
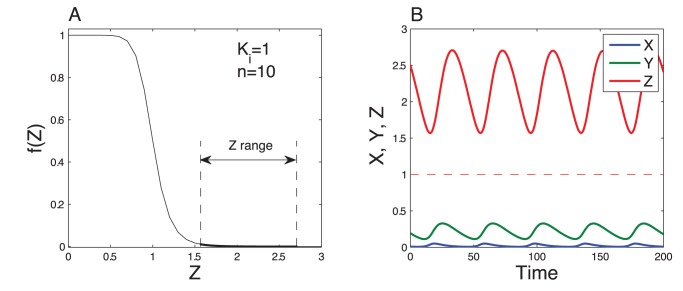
Dynamics of Goodwin model. (A) Inhibitory Hill function (Eq. (4), with 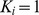 and 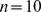). (B) Limit cycle oscillations obtained by numerical integration of Eqs. (1)–(3) for the following parameter values (arbitrary units): 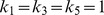, 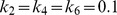, 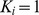, 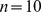. The oscillation period is about 40 a.u. The dashed line indicates the Hill threshold 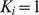.

The Hill function and the limit-cycle oscillations obtained by numerical integration of the Goodwin model are shown in Fig. 2. For the parameter values chosen (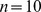), 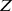 remains always above the threshold 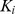 and the oscillations are rather sinusoidal. When 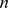 increases, we can make 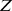 switching around 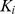, inducing relaxation oscillations [13]. The period of the oscillations can be easily adjusted to a circadian value by a proper rescaling of the time-dependent parameters.

In the following, our primary goal is to replace the Hill function in Eq. (1) by a realistic molecular mechanism, namely a multisite phosphorylation process, and to show that the Hill function is a proper description of the kinetics of this mechanistic model, at least under some conditions.

### Multisite Phosphorylation

Post-translational modifications are crucial for the dynamics of biochemical systems. By inducing conformational changes, protein phosphorylation can regulate the catalytic or transcriptional activity of the protein in a fast and efficient way. About 30% of the proteins of an eukaryotic cell undergo phosphorylation, often on several sites [32]. Phosphorylation on multiple sites of a protein was reported in signaling pathways [32], but also in genetic circuits, including circadian clocks [34], [35] and the cell cycle [36], [37]. By generating sharp thresholds, multisite phosphorylation can induce multiple steady states or favor the emergence of oscillations. Multisite phosphorylation provides also a means to adjust robustly the timing of cellular events [38].

We show here that Hill-like response curves can be readily obtained in a multisite phosphorylation mechanism. [2] derived a general expression for the kinetics of the phosphorylation/dephosphorylation rates of a protein, in the case of ordered multisite phosphorylation:
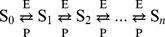



In this scheme, a protein S, with 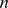 sites, can be found in various forms 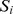, where 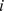 is the number of phosphorylated sites. Each phosphorylation step (catalyzed by a kinase E) and dephosphorylation step (catalyzed by a phosphatase P) are assumed to follow a Michaelis-Menten mechanism:
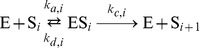
(5)

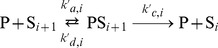
(6)for 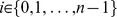. The time evolution of the concentration of 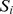, 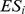, and 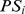 are described by the following 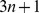 kinetic equations:
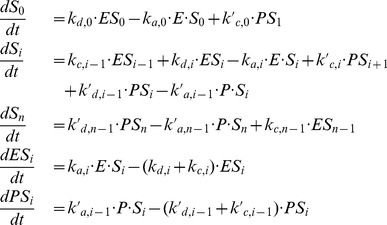
(7)for 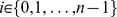. The total concentrations of protein S (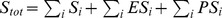), kinase E (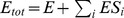) and phosphatase P (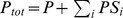) are constant, leading to 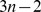 independent equations.

Gunawardena (2005) then showed that the fraction of maximally phosphorylated protein.
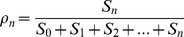
(8)can be approximated by the Hill function:
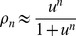
(9)with 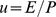, providing some constraints on the kinetic parameters. 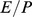 can be further approximated by 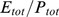 with some conditions on 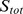, 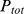 and 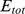. The detailed calculations and the conditions on parameter values can be found in Gunawardena [2] and are summarized in Supplementary Material (Text S1, Section A and Fig. S1).

Since 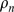 takes into account only the free forms of 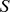, this measure may not be appropriate to describe, for example, the effective concentration of a transcription factor, as described later. A more suitable fraction would be:
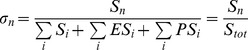
(10)


Following Gunawardena’s work, we derived constraints on the parameter values to approximate 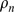 by 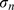 and alternative constraints to those derived by Gunawardena to approximate 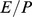 by 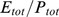 (see Text S1, Section B and Table S1). Notably, these constraints do not impose small ratio of 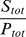 contrary to Gunawardena’s conditions. In contrast, a large difference in the kinetics of the last phosphorylation/dephosphorylation is sufficient. More specifically, in our simulations we choose, for the last dephosphorylation step a rate much faster than in the previous dephosphorylation steps. Quantitative experimental data on phosphorylation/dephosphorylation rates are very poor and we did not find any evidence for such “last-step” cooperativity effect. Since phosphorylation may affect the conformation and the binding ability of proteins, it is not inconceivable that the fully phosphorylated form of the protein affects its kinetics. This does not exclude the possibility that other sets of parameter values also lead to a good agreement.

**Figure 3:**
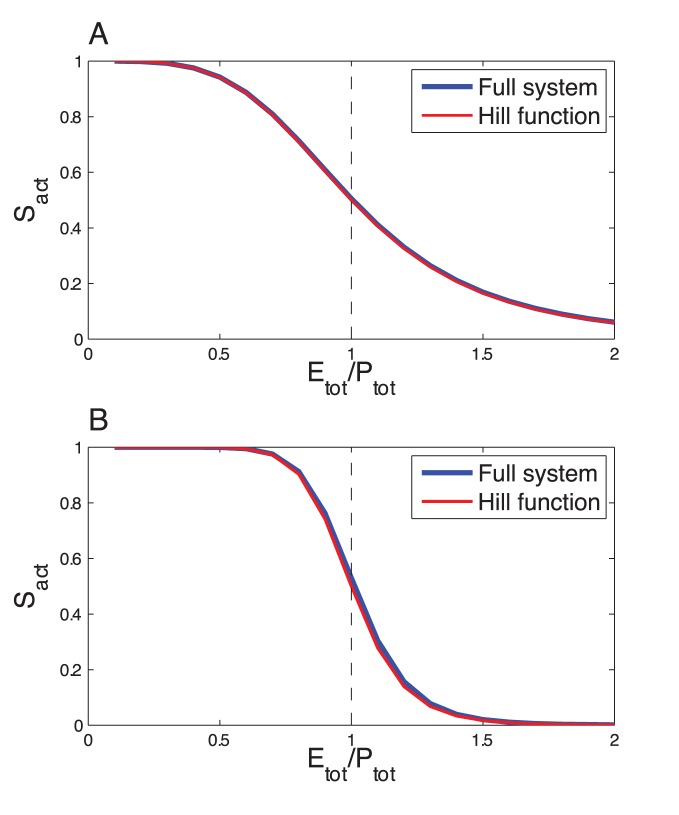
Multisite phosphorylation. Comparison of the response curve of the full system and the Hill function for (A) 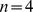 and (B) 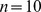. The blue curve gives the steady state value of 
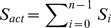
 as a function of the ratio 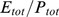, obtained by numerical integration of Eqs. (7). The parameter values are: 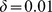 (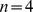), 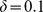 (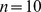), 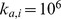, 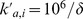, 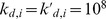, 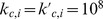 (for 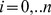), except 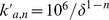 (for the last dephosphorylation step), 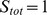, 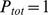, 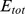 varies from 0 to 2. For the Hill function (red curve), 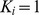.

Using theses constraints as a guide to tune the parameter values, we successfully found parameter values that yield a steady state response curve of 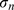 as a function 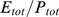 that accurately fits the Hill curve, both for 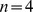 using a 4-site phosphorylation system (Fig. 3A) and for 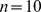 using a 10-site phosphorylation system (Fig. 3B). The kinetic constants satisfy the constraints derived by Gunawardena. In particular, the kinetic parameter of the phosphorylation and the dephosphorylations steps are the same, except for the last step. Remark that we plotted 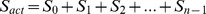. In the following, we will always take 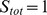, so that 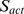 represents as well the absolute or relative concentration of the active form of the protein.

This result confirms that, upon appropriate assumptions, multisite phosphorylation can lead to Hill-like response curve. Note that other response curves, with more/less sharp thresholds can be obtained with other sets of parameter values (see for example the case of ultrasensitivity discussed here below), but they can not always be accurately approximated by Hill functions. We thus have at hand a realistic molecular mechanism producing Hill-like kinetics, which can now be integrated in the Goodwin model.

### Combining the Multisite Phosphorylation and the Goodwin Model

We now propose a mechanistic description of the Goodwin model in which the Hill function is replaced by the multisite phosphorylation module described in the previous section.

**Figure 4:**
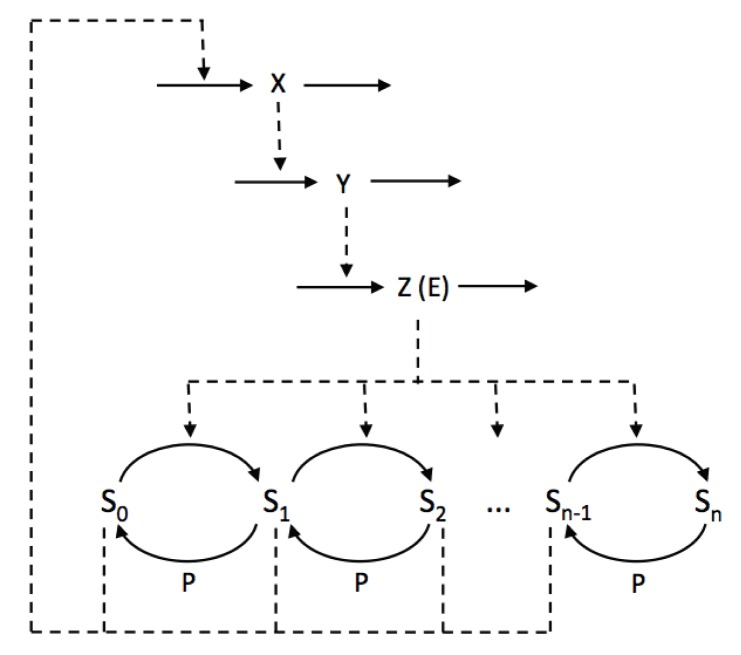
Scheme of the Goodwin model combined with the multisite phosphorylation module. In this model, variable 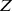 is the kinase E which can be found in its free form or in a complex with any phosphoform of S. We also assume that all forms of free S, except 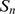, can induce the synthesis of X.

We will assume that (1) the Goodwin variable 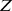 is the total concentration 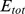 of a kinase E that catalyzes the sequential phosphorylation of a transcription factor S on 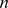 sites, (2) all free forms of S except the fully phosphorylated form (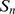) are equally active to induce the transcription, i.e. the synthesis of X, and (3) 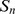 does not induce the transcription of X (Fig. 4).

The complete model is described by the following 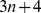 equations:
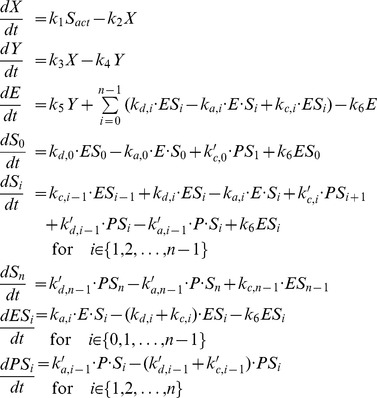
(11)where



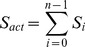
(12)In this model, we also assume that the enzyme E, being in its free form (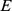) or in a complex with any phosphorylated form of S (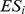, with 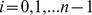), is degraded at the same rate 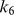. To keep the total concentration of S, 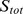, constant, we further assume that when the complex ES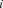 is “eliminated”, E is actually degraded and the free form of S, 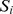 is released. Given these assumptions and under the hypothesis that the kinetics of the phosphorylation module occurs at a much faster time-scale than the rest of the system (quasi-steady state assumption), the model can actually be reduced to the 3-variable Goodwin model, with 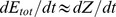 (see Text S1, Section C, for the mathematical derivation).

**Figure 5:**
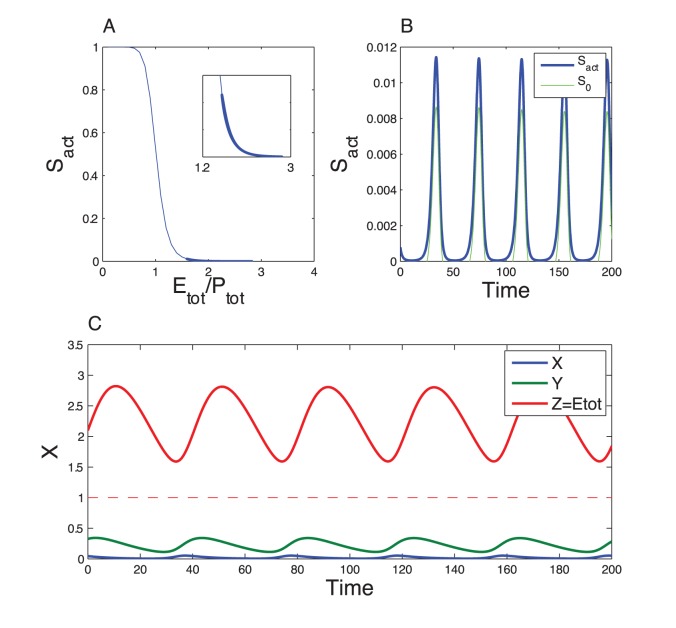
Dynamics of the Goodwin model combined with the multisite phosphorylation module. These results have been obtained by numerical integration of Eqs. (11). Parameter values for the Goodwin model are as in Fig. 2. Kinetic parameter values for the phosphorylation module are as in Fig. 3 for 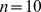 but multiplied by a factor 100. Conservation parameter values, 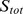 and 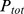, are the same as in Fig. 3. In panel A, the thin blue curve corresponds to 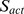 at steady state, as obtained in Fig. 3, while the thick line denotes the trajectory of the present system. The inset is a zoom on the lower part of that curve. The oscillations are in very good agreement with the oscillations generated by the 3-variable model (Fig. 2, see also Fig. 7 for a comparison of the limit cycles). The period of the oscillations, about 40 a.u., is also consistent with the 3-variable model.

**Figure 6:**
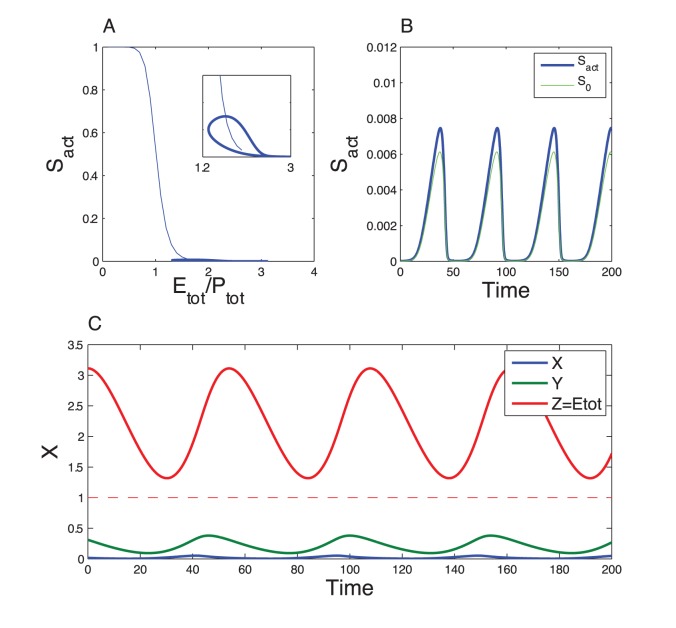
Effect of the speed of the phosphorylation module. The results have been obtained for the same equations as in Fig. 5. The kinetic parameter values of the phosphorylation module have been decreased by a factor 100 compared to Fig. 5. The other parameter values are the same as in Fig. 5. The trajectory of the complete system does not match the steady state curve because the dynamics of 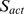 is not sufficiently fast (Panel A). Nevertheless the oscillations are not significantly affected compared to the 3-variable model (see Fig. 7 for a comparison of the limit cycles). Due to a slowing down of the dynamics, the period (54 a.u.) is longer than the oscillation period in the 3-variable model.

**Figure 7:**
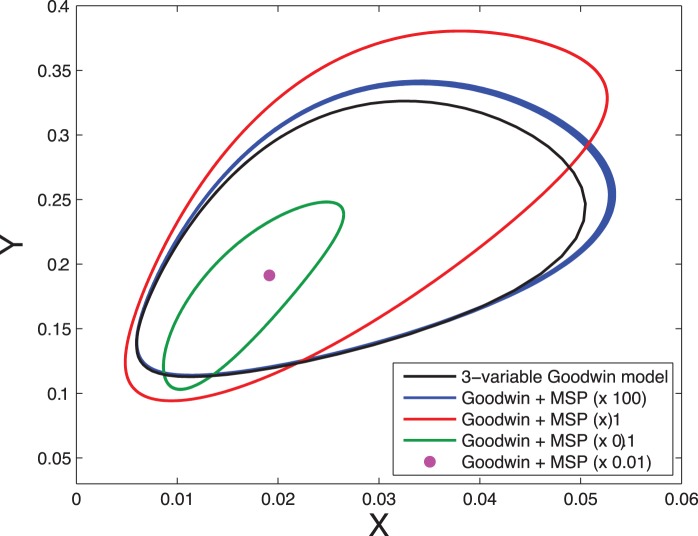
Comparison of the 3-variable Goodwin model with its variant based on multisite phosphorylation. Shown are the limit cycles obtained for the 3-variable model (black curve) and for its variant based on multisite phosphorylation, for various speeds of the phosphorylation module. The blue and red curves correspond to the cases illustrated in Figs. 5 and 6, respectively. The green curve is the limit cycle obtained when the kinetics rates of the multisite phosphorylation module are decreased by a factor 10 compared to the values given in Fig. 3. The violet dot is the stable steady state of the system when the kinetics rates of the multisite phosphorylation module are reduced by a factor 100.

To assess the validity of the quasi-steady state assumption, we performed numerical simulation of the complete model (Eqs. (11)), for 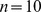. Figure 5 shows the dynamics of the model when the kinetics of the phosphorylation/dephosphorylation reactions are fast compared to the dynamics of the Goodwin variables. The oscillations obtained are in very good quantitative agreement with the prediction of the three variable model. When the phosphorylation/dephosphorylation reaction rates are reduced by a factor 100, limit-cycle oscillations still occur (Fig. 6) but their amplitude and period slightly depart from the limit-cycle generated by the 3-variable model (Fig. 7, red and black curves). If the reaction rates are decreased by a factor 1000, the oscillations are even more altered and their amplitude is significantly reduced (Fig. 7, green curve). Finally, if the reaction rates are further decreased, by a factor 10000, oscillations are lost and the system converges to a stable steady state (Fig. 7, violet dot). Using Michaelis-Menten kinetics instead of mass action laws for each phosphorylation/dephosphorylation step leads to a very good agreement with the above detailed model (See Text S1, Section D and Fig. S2).

In summary, the Hill function in the Goodwin model can be justified by the quasi-steady state limit of a multisite phosphorylation mechanism of a transcription factor. Constraints on the speed of the phosphorylation module have however to be fulfilled to make the quasi-steady state assumption valid. In particular the speed of these post-translational modification must be fast compared to the other processes of the oscillatory mechanism, such as the synthesis and degradation rates. These constraints add to those on the kinetic parameters which allow a Hill-like response of the phosphorylation module (see previous section and Table S1).

### Zero-order Ultrasensitivity

Multisite phosphorylation is not the only way to generate sharp thresholds. In fact, a single, reversible, phosphorylation mechanism can, under some conditions, produce very abrupt thresholds [5]. This so-called zero-order ultrasensitivity effect was used in a number of computational models in biology, namely in models for the cell cycle [39], [40]. The derivation of the kinetics equations for zero-order ultrasensitivity is provided in Text S1 (Section E), Fig. S3, and Table S1.

**Figure 8:**
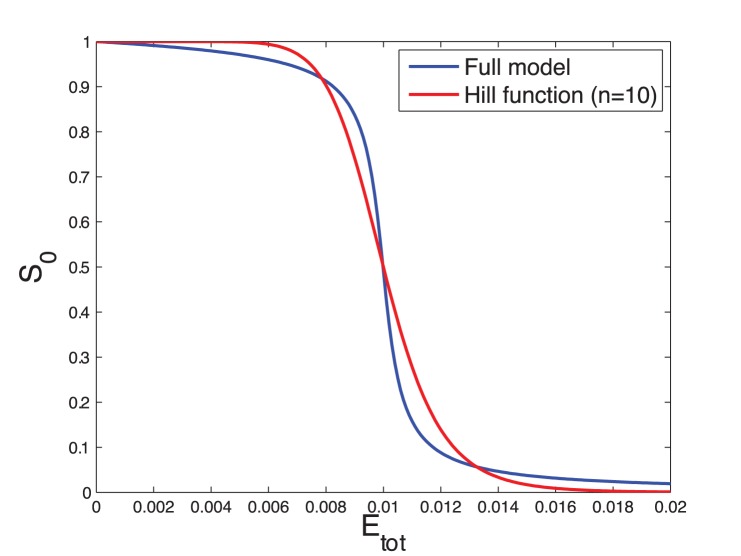
Zero-order ultrasensitivity. Comparison of the response curve of the phosphorylation/dephosphorylation module (Eqs. (S44), equivalent to Eqs. (7) with 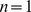) and the Hill function. The parameter values are: 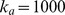, 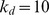, 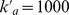, 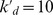, 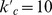, 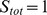 and 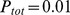. The blue curve corresponds to the steady state value of 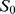 as a function of the total concentration of the kinase, 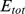 (cf Text S1, Section D). The red curve is the Hill function plotted for 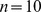 and 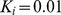. Note that a Hill curve with 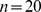 better fits the middle part but not the upper and lower parts of the response curve of the phosphorylation/dephosphorylation module (not shown).

We could not find parameter values for which the response curve of the “zero-order ultrasensitivity” function tightly fits the Hill function. There is actually a trade-off to set between the fitting of the middle part and the fitting of the upper and lower part of the response curve (Fig 8). We therefore manually tuned the kinetic parameters of the zero-order ultrasensitivity module such that its response curve approximates Hill function (with given Hill coefficient), using conditions derived in Text S1 (Section E) as an initial guess for this parameter setting (Fig. 8).

**Figure 9:**
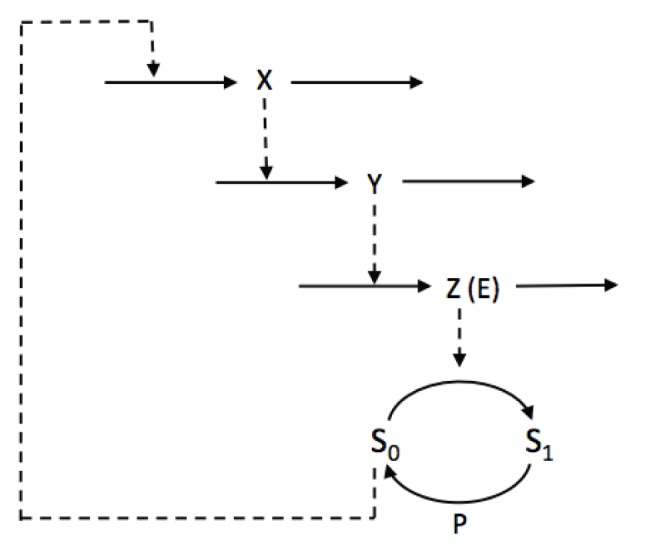
Scheme of the Goodwin model combined with a single phosphorylation/dephosphorylation module. The phosphorylation/dephosphorylation module can generate sharp thresholds through “zero-order ultrasensitivity”.

The Hill function of the Goodwin model was then replaced by this mechanism (Fig. 9). As before, we will assume that Z is the kinase E and that only the dephosphorylated form of S, 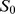 (equivalent to 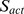 in the previous model), activates the synthesis of X. The model is thus the same as the previous one, for the particular case where 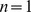.

The time evolution of the model is governed by the following 7 equations:
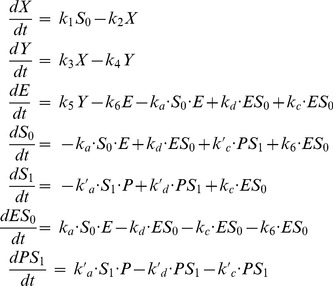
(13)


**Figure 10:**
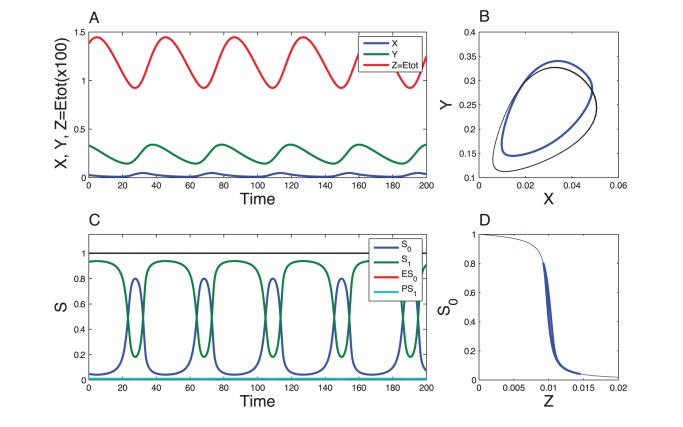
Dynamics of the Goodwin model combined with a “zero-order ultrasensitivity” phosphorylation module. The time series have been obtained by numerical integration of Eqs. (13), with the following parameter values: 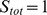, 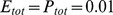, 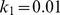, 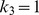, 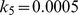, 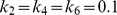. The kinetics rates of the phosphorylation/dephosphorylation reactions are as in Fig. 8 but multiplied by a factor 100. For clarity and to facilitate the comparison with the 3-variable model, variable 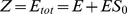 has been multiplied by a factor 100. In panel B, a comparison of the limit cycle obtained for the present model (blue curve) and for the original 3-variable Goodwin model (black curve) shows a reasonable agreement between the two models. The period, about 40.7 a.u., is very close the period of the original Goodwin model. In panel D, the thin curve corresponds to the steady state of the phosphorylation module (cf. Fig. 8) and the thick curve is the trajectory of the present system.

The system defined by Eqs. (13) can generate limit-cycle oscillations, which are in reasonable agreement with the prediction of the original 3-variable model (Fig. 10). To obtain such quantitative agreement, we had (1) to set the system in zero-order ultrasensitivity conditions, (2) to assume that the kinetics of the phosphorylation module is much faster than the kinetics of the rest of the system, and (3) to (manually) adjust some kinetic parameters.

A few other differences with the previous model are worth mentionning. First, contrary to the case of multisite phosphorylation, one condition to obtain zero-order ultrasensitivity in a single phosphorylation cycle is that the substrate S is in high quantity with respect to the enzymes (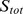 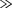 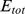, 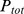) (see Table S1). We therefore had to decrease the scale of the variable 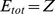 (by a factor 100 in our simulation) compared to the 3-variable model. Second, as shown in Fig. 10C, the fraction of active S (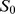) reaches much higher amplitude than in the multisite phosphorylation model because the threshold effect is here more pronounced (Fig. 10D).

As observed in the multisite phosphorylation model, limit cycle oscillations are lost when the phosphorylation/dephosphorylation reactions are too slow. On the contrary when the kinetics of the phosphorylation/dephosphorylation reactions is very fast, limit cycle oscillations are preserved and match their quasi-steady state approximation (Eqs. S58-S60) (not shown). We also checked that using Michaelis-Menten kinetics instead of mass action laws leads to consistent results (not shown).

In summary, zero-order ultrasensitivity arising in single reversible phosphorylation mechanism provides an efficient way to generate sharp thresholds. Although it does not lead to a perfect quantitative fit with the 3-variable Goodwin model, it constitutes a very simple mechanism which can be used to substitute for the Hill function in the Goodwin model to generate limit-cycle oscillations.

### Phase-resetting Goodwin Model

Ruoff et al [20], [21] introduced a variant of the Goodwin model in which the Hill function is replaced by an arbitrary 2-threshold “reset function”, 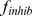, which is set to 0 when 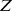 crosses upwards the value of 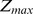 and to 1 when 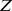 crosses downwards the value of 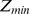.

The equations for this system write:
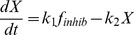
(14)

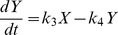
(15)

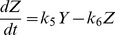
(16)


**Figure 11:**
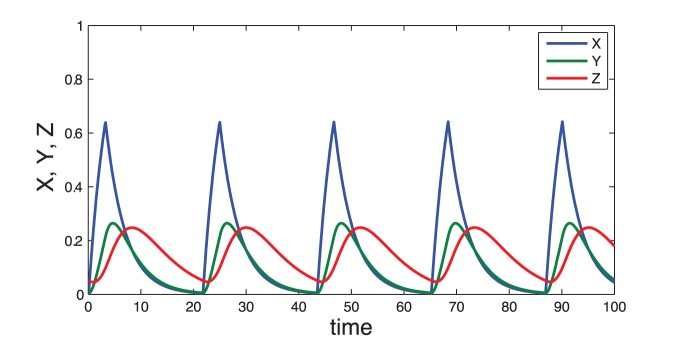
Dynamics of the “reset” Goodwin model. The time series have been obtained by numerical integration of Eqs. (14)-(16) for the following parameter values: 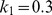, 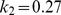, 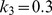, 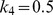, 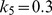, 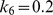, 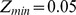 and 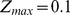 (values taken from [21]). Oscillations have a period of about 24 a.u.

This phase-resetting model produces relaxation oscillations (Fig. 11) and was used to reproduce phase response curves [20] and to study temperature compensation [21] in circadian systems.

We seek here to find a realistic molecular mechanism that can account for these abrupt phase resettings. Intuitively, a mechanism based on bistability should explain such double threshold effect and the rapid switch between the two states of the reset function.

### Bistability Arising from a Double Phosphorylation Cycle

Bistability, defined as the coexistence of two stable steady states, is thought to constitute the central process of cellular differentiation [41], but appears to be also involved in the molecular mechanism of cellular oscillators, such as the cell cycle [42]. Kholodenko and coauthors [33], [43], [44] showed that bistability can occur in a system involving two reversible phosphorylations, provided that some conditions on kinetic parameters are fulfilled.

We consider here the double phosphorylation model described in Ortega et al [43], whose detailed version is equivalent to the model defined by Eqs. (7) for the case 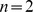. The variables 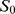, 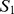, and 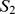 represent the different forms of a protein, namely the non-phosphorylated form 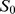, the mono-phosphorylated form 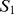, and the bi-phosphorylated form 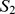. The total level of the protein is constant: 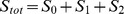. The phosphorylations are supposed to take place in a processive way.

The kinetic equations for this double phosphorylation model are:
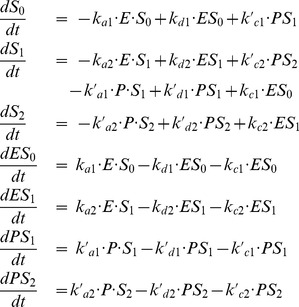
(17)where the total concentration of kinase (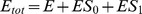) and phosphatase (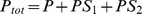) are constant.

**Figure 12:**
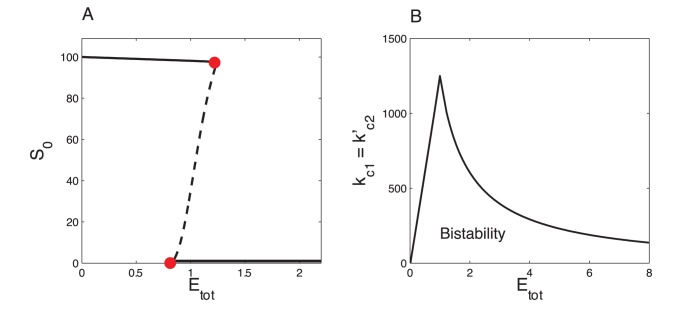
Bistability in the double-phosphorylation system (Eq. (17)). (A) Bifurcation diagram showing the steady state of 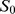 as a function of 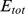. (B) Bistability domain in the 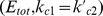 parameter space. The other parameter values are: 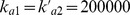, 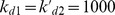, 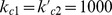, 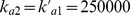, 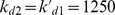, 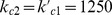, 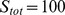 and 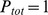. The bistable domain in panel A is delimited by two saddle-node bifurcation points at 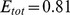 and 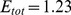 (red points). The simulations have been carried out using XPP-AUTO software [49].

Ortega et al. [43] derived conditions on the parameter values to set the system in a bistable domain (see Text S1, Section F, and Table S1). The parameter values of our model were thus adjusted according to these constraints and numerical simulations of the system were performed. The result of the simulation shows a bistable domain, in a bounded window of values of 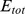, delimited by two saddle node bifurcation points (Fig. 12). Note that, outside the bistable regime, 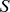 is either mainly in the free form (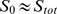) or mainly in the biphosphorylated form (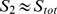). The intermediary form 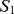 is always present in very low quantity.

**Figure 13:**
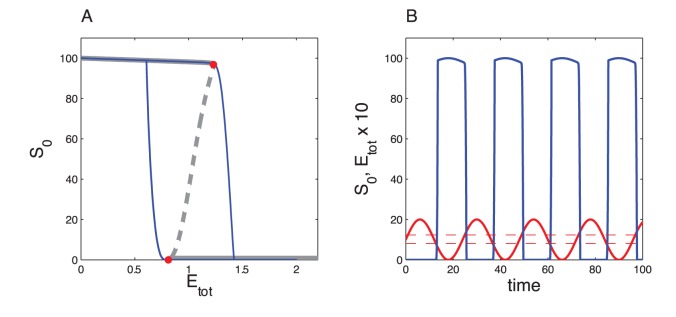
Effect of a periodic modulation of 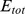 on the bistable system of the double-phosphorylation module. In panel A, the grey curve represents the steady state of 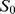 as a function of 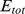 and the blue curve the trajectory of the system. In panel B, the blue curve is 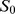. The parameter values are the same as in Fig. 12. 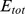 follows a sine wave with period of 24 a.u. and amplitude of 2 a.u. (red curve).

We then check that this bistable model can, in response to a periodic signal, reproduce the abrupt switches reminiscent of the arbitrary resetting created by the 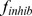 function. To do so, we examined the effect of a periodic modulation of 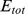 on the bistable module (Fig. 13). The results confirm that if the kinetics of the bistable system is sufficiently fast the bistable module produces abrupt switches, which occur when 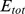 reaches the saddle node bifurcation points.

### Combining the Bistable Module and the Resetting Goodwin Model

**Figure 14:**
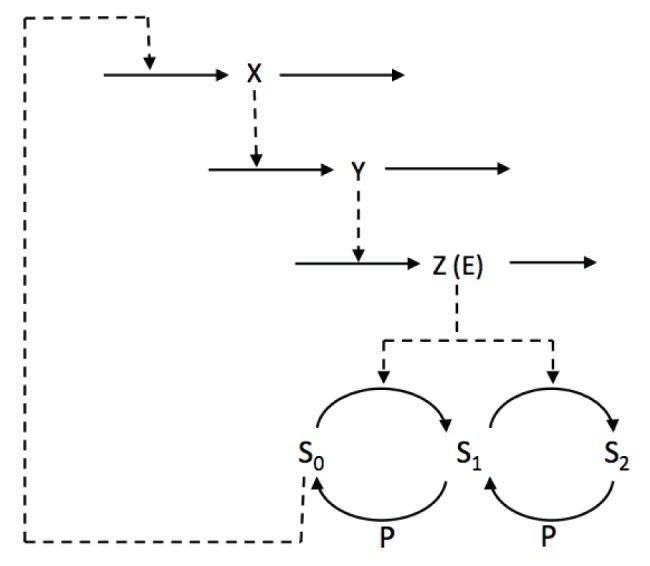
Scheme of the Goodwin model combined with the double-phosphorylation module producing bistability.



In a second step, we plugged the bistable module in the Goodwin system, thereby replacing the 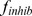 function by the variable 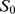 (Fig. 14). As in the previous models, we will also assume that Z is the kinase E. The complete model has 10 variables, whose evolution is governed by the following equations:

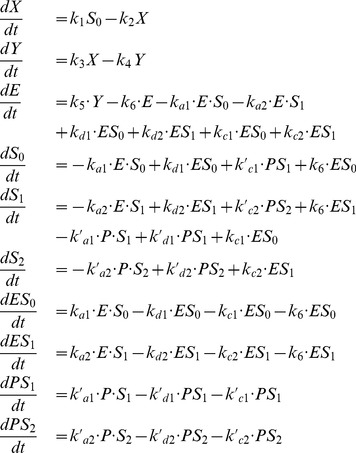
(18)with 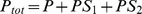 is constant and 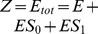 evolves with time.

To obtain a good agreement between the present model and the 3-variable model, we (manually) adjusted the parameters values in order to roughly set the bifurcation points (delimiting the bistable domain) to 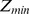 and 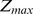. This was achieved by rescaling 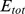 by a factor 10 (through the constant 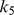) and 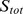 by a factor 100 (through 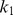).

**Figure 15:**
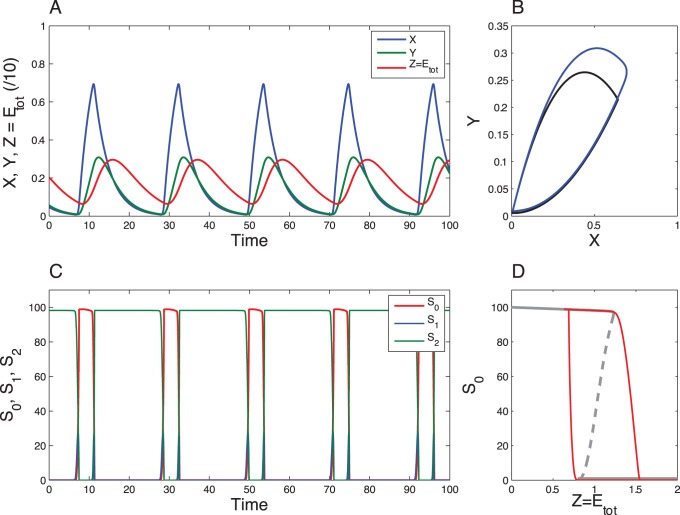
Dynamics of the Goodwin model coupled to the bistable module (Eqs (18)). The parameter values for the Goodwin module are as in Fig. 11 except 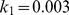 and 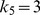. The parameter values for the bistable module are as in Fig. 13. In panel B, the black curve represents the limit cycle for the 3-variable model while the blue curve is the limit cycle for the present model. In panel D, the grey curve represents the steady state of the bistable module (as in Fig. 13) and the red curve is the trajectory of the present system.

For this parameter setting, numerical simulation of this system generates relaxation oscillations similar to the ones obtained with the “2-threshold” Goodwin model (Fig. 15). We can also observe that 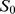 undergoes very abrupt switches between 0 and 100, mimicking the effect of 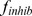.

Thus, these results provide a rational molecular mechanism for the arbitrary resetting function proposed by Ruoff (2001, 2005). We can also anticipate that any system displaying bistability could, upon appropriate calibration, generate similar switch-like effects.

## Discussion

The phenomenological Goodwin model constitutes a generic oscillator model in biology. It was originally developed to demonstrate the occurrence of oscillations in a delayed negative feedback loop system [8]. It contains only 3 variables and involves a single non-linear term, which takes the form of a Hill function. Analytical studies of this system demonstrate that limit-cycle oscillations can be produced by this model if the Hill coefficient 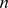 is greater than 8 [9]. Such high non-linearity is hard to justify mechanistically through realistic cooperative processes which arise in enzymatic kinetics or at a transcriptional level. More recently, a variant of the Goodwin model, in which the Hill function has been replaced by an arbitrary 2-threshold “reset” function [20], [21], has been proposed to model circadian systems. The goal of the present paper was to examine alternative molecular mechanisms that may explain this sharp threshold kinetics and thereby validate the use of the standard and the phase-resetting Goodwin models for biochemical systems. We focused on phosphorylation/dephosphorylation of a single transcription factor and studied three molecular mechanisms which have been shown to produce step-like responses: a multisite phosphorylation/dephosphorylation, a double phosphorylation/dephosphorylation (giving rise to bistability) and a single phosphorylation/dephosphorylation mechanism (producing zero-order ultrasensitivity).

A genome-scale screening of enrichment of phosphorylated proteins revealed that about one third of the proteins undergo phosphorylation, most often on multiple sites [32]. Several theoretical studies have shown that sharp thresholds can easily be generated from such post-translational modifications [2], [3], [26]–[29]. Multisite phosphorylation has been shown to play a key role in the regulation of signalling pathways but also in genetic networks, including circadian clocks [34], [35] and the cell cycle [36], [37]. By inducing conformation changes in proteins, multisite phosphorylation provides an efficient way to control the catalytic activity of enzymes or the regulatory activity of transcription factors [45].

We have shown here that a Hill-type response curve can result from a multisite phosphorylation of a transcription factor and that such mechanism can substitute to the arbitray Hill function in the Goodwin model. The Goodwin model combined to a multisite phosphorylation process yields limit-cycle oscillations which are in very good quantitative agreement with the limit-cycle oscillations generated by the 3-variable model. This matching is obtained when the speed of the phosphorylation module is fast compared to the rest of the system and under some additional conditions on the parameter values. In particular, disparate kinetics rates are required. In these conditions, the quasi-steady state approximation can be applied and the Goodwin model can be seen as a limit case of the full system based on multisite phosphorylation when the kinetics of the phosphorylation module is fast. Even if the perfect agreement would only be reached for infinitely fast phosphorylation/dephosphorylation rates, the Goodwin model remains a good and convenient approximation of the complete system. This reduced model contains only 3 variables whereas the full system (for 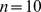) counts 34 variables. More importantly, the CPU time to simulate the 3-variable system is significantly smaller than for the full system because the latter involves a mixture of slow and fast reactions, requiring stiff integration methods.

To generate sharp threshold, it is not necessary to resort to multisite phosphorylation processes. Actually, a single, reversible phosphorylation/dephosphorylation mechanism can already produce ultrasensitivity, provided that the Michaelian constants are very low [5]. The response curve obtained in that case, however, can not be accurately approximated by a Hill kinetics. We showed that such process can nevertheless be integrated in the Goodwin model and, after adjusting some kinetic parameter values, generates limit-cycle oscillations which match reasonably well the ones obtained in the original 3-variable model.

Bistable switches provide another means to produce abrupt transition. Bistability behaviour can be induced by a double phosphorylation/dephosphorylation mechanism [33], [43], [44]. We have shown here that such molecular description provides a realistic mechanism consistent with the arbitrary reset function used by Ruoff et al [20], [21]. In this way, relaxation oscillations can be generated easily, without requiring an additional, positive, transcriptional feedback loop, or a Hill process with very high Hill coefficient [13].

The Goodwin model can thus be seen as a transcription-translation feedback loop, coupled to a post-translational mechanism. We should stress that, beyond the particular case of the Goodwin model, the proposed mechanism supports the use of Hill functions with moderate Hill coefficient (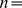
4 or 5) as postulated in many genetic models [17]–[19]. It is also worth mentionning that the response curve of the phosphorylation modules does not necessarily need to closely fit the Hill function to obtain limit-cycle oscillations. We focus here on Hill function because it is commonly used in biological systems. Of course, we do not exclude that other post-translational processes could lead to similar kinetics and could provide alternative, suitable support for the Goodwin (and related) model.

The dynamics of post-translational processes is often studied at the steady state level. Properties like response curves or bistability reflect static features of these systems. However signalling pathways and genetic network rarely operate at steady state. It is therefore important to assess the behaviour of these post-translational regulations when they are embedded in a dynamical system, such as a genetic oscillator. Our results highlight the impact of the speed of phosphorylation/dephosphorylation as slow phosphorylation process can significantly alter the oscillatory behavior of the detailed Goodwin model. The assumption that the binding/unbinding of the kinase (or phosphatase) to the regulatory protein is much faster than transcription and translation processes is in principle reasonable. Changing gene expression can take hours and even days while posttranslational protein modifications usually take minutes to occur. However, this assumption on time scale separation should be discussed in specific contexts.

Ultrasensitivity and multisite phosphorylation have already been exploited to construct minimal and detailed models for the cell cycle [37], [39], [46], [47] and for circadian clocks [18], [48]. Here, we have shown that the Goodwin model may be used as a building block to model a large class of biological oscillators based on the negative transcription feedback loop coupled to post-translational modifications.

## Materials and Methods

Temporal simulations and responses curves have been performed with Matlab (using the *ode23tb* integrator for the detailed models). Response curves (Figs. 3 and 8) have been obtained by running numerical simulations until the system converged to its steady state, which is then recorded for various values of the control parameter (usually 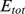). Bifurcation diagrams shown in Figs. 12 and 13 have been generated with XPP-AUTO [49].

## Supporting Information

Figure S1Multisite phosphorylation vs Hill approximation. Response curves obtained by the multisite phosphorylation mechanism (blue dashed curves) are compared to Hill functions (blue curve), for 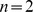, 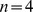, and 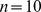 (see also Text S1, Section A).(EPS)

Figure S2 Michaelis-Menten approximation. Comparison of the limit cycle obtained by the fully detailed Goodwin model combined to the multisite phosphorylation module (blue curve) and the limit cycle obtained using the Michaelis-Menten equations (red curve) (see also Text S1, Section D). (EPS)

Figure S3Zero-order ultrasensitivity. Response curve of 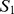 as a function of the ratio 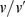 (as defined by Eq. (S59)), plotted for various values of 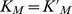 and 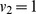 (see Text S1, Section E).(EPS)

Table S1 Parameter conditions required for the derivation of the response curves for the mechanistic modules studied in this work. The definition of the parameters and the derivation of the conditions established by Gunawardena, Goldbeter and Koshland, and Ortega et al. can be found in supplementary material section A, E and F respectively. For more details about the derivation, see the original papers. The equations are taken at steady state for the derivation of all the conditions. Note that, as mentionned in the text, approximating the Goodwin model combined with each mechanistic module with the Goodwin model requires additional conditions on the time scales of the kinetic parameters, i.e. the kinetics of the mechanistic phosphorylation module has to be much faster than the kinetics of the rest of the model (Quasi-steady state approximation conditions). (PDF)

Text S1 Supporting Material. (PDF)
