# Supplementary material for: The Goodwin Model: Behind the Hill Function
Source: PLoS One. 2013 Aug 1;8(8):e69573. doi: 10.1371/journal.pone.0069573 (PMC3731313; doi:10.1371/journal.pone.0069573)
Supplement: Table S1 — (PDF) [file pone.0069573.s004.pdf]

MODULES	RESPONSE CURVE	PARAMETER CONDITIONS	REFERENCE
**Multisite phosphorylation**	Hill function (Hill coefficient n)	$\lambda_0 = \lambda_1 = ... = \lambda_{n-2} = \delta$<br>$\lambda_{n-1} = \delta^{1-n}$<br>$\delta \ll 1$	Gunawardena (2005)
		$S_{tot} \ll K_{Mmin}, K'_{Mmax}$<br>$E_{tot} \ll K_{Mmin}$<br>$P_{tot} \ll K'_{Mmin}$	Supplementary material, section B
**Single site phosphorylation**	Zero-order ultrasensitivity	$S_{tot} \gg E_{tot}, P_{tot}$<br>$K_M, K'_M \ll 1$	Goldbeter and Koshland (1981)
**Double site phosphorylation**	Bistable	$S_{tot} \gg E_{tot}, P_{tot}$<br>$K_{M1} = K_{M2} = K'_{M1} = K'_{M2}$<br>$\theta > \frac{(1+K_S)^2}{(1-2K_S)^2}$<br>$K_S > \frac{1}{2}$	Ortega et al. (2006)
