# Supplementary material for: The Goodwin Model: Behind the Hill Function
Source: PLoS One. 2013 Aug 1;8(8):e69573. doi: 10.1371/journal.pone.0069573 (PMC3731313; doi:10.1371/journal.pone.0069573)
Supplement: Text S1 — (PDF) [file pone.0069573.s005.pdf]

Supplementary Material

The Goodwin model: Behind the Hill function

Didier Gonze[1] and Wassim Abou-Jaoudé[2]

(1) Faculté des Sciences, Université Libre de Bruxelles, Bruxelles, Belgium. (2) Institut de Biologie, Ecole Normale Supérieure, Paris, France.

June 17, 2013

Content

A. Multisite phosphorylation: Convergence of ρ_n to the Hill function

B. Multisite phosphorylation: Convergence of σ_n to the Hill function

C. Goodwin model and multisite phosphorylation: Quasi-steady state assumption

D. Goodwin model and multisite phosphorylation: Michaelis-Menten kinetics

E. Zero-order ultrasensitivity in the single site phosphorylation system

F. Bistability in the 2-site phosphorylation system

A. Multisite phosphorylation: Convergence of ρ_n to the Hill function

We summarize here the derivation of the kinetics equations for a multisite phosphorylation system and show in which conditions the response curve ρ_n as a function of $\frac{E}{P}$ can converge to a Hill function (see Gunawardena, (2005) for details). These conditions are summarized in Table S1.

The reaction schemes of the phosphorylation/dephosphorylation steps are:

$$\mathrm{E} + \mathrm{S}_i \underset{k_{d,i}}{\overset{k_{a,i}}{\rightleftharpoons}} \mathrm{ES}_i \overset{k_{c,i}}{\rightarrow} \mathrm{E} + \mathrm{S}_{i+1} \tag{S1}$$

$$\mathrm{P} + \mathrm{S}_{i+1} \underset{k'_{d,i}}{\overset{k'_{a,i}}{\rightleftharpoons}} \mathrm{PS}_{i+1} \overset{k'_{c,i}}{\rightarrow} \mathrm{P} + \mathrm{S}_i \tag{S2}$$

for $i \in \{0, 1, \ldots, n-1\}$. At steady state, the net flux of each phosphorylation/dephosphorylation step must be null, leading to the following relations:

$$k_{c,i} ES_i = \frac{k_{c,i}}{K_{Mi}} E \cdot S_i \tag{S3}$$

$$k'_{c,i} PS_{i+1} = \frac{k'_{c,i}}{K'_{Mi}} E \cdot S_i \tag{S4}$$

where $K_{Mi} = (k_{c,i} + k_{d,i})/k_{a,i}$ and $K'_{Mi} = (k'_{c,i} + k'_{d,i})/k'_{a,i}$ are the Michaelian constants. Gunawardena (2005) then showed that the fraction of maximally phosphorylated protein

$$\rho_n = \frac{S_n}{S_0 + S_1 + S_2 + ... + S_n} \tag{S5}$$

can be expressed as a function of $u = E/P$ by:

$$\rho_n = \frac{\lambda_0 \lambda_1 ... \lambda_{n-1} u^n}{1 + \lambda_0 u + \lambda_0 \lambda_1 u^2 + ... + \lambda_0 \lambda_1 ... \lambda_{n-1} u^n} \tag{S6}$$

where $\lambda_i = \frac{k_{c,i}/K_{Mi}}{k'_{c,i}/K'_{Mi}}$ must satisfy, at steady state:

$$\frac{S_{i+1}}{S_i} = \lambda_i \frac{E}{P} \tag{S7}$$

Choosing λ_i such that:

$$\lambda_0 = \lambda_1 = ... = \lambda_{n-2} = \delta \text{ and } \lambda_{n-1} = \delta^{1-n} \tag{S8}$$

and taking small δ value, Eq. (S6) is well approximated by a Hill-like curve (Fig. S1):

$$\rho_n = \frac{u^n}{1 + (\delta u) + (\delta u)^2 + ... + (\delta u)^{n-1} + u^n} \approx \frac{u^n}{1 + u^n} \tag{S9}$$

Note that Gunawardena (2005) does not provide numerical values for the kinetic constants k_a, k_b, k_c, etc.

B. Multisite phosphorylation: Convergence of σ_n to the Hill function

We look here for conditions on parameter values that leads to Hill-like response curve of σ_n as a function of $\dfrac{E_{tot}}{P_{tot}}$ in the case where $S_{tot} = P_{tot}$. We recall the expressions of ρ_n and σ_n:

$$\rho_n = \frac{S_n}{S_0 + S_1 + \ldots + S_n}$$
$$\sigma_n = \frac{S_n}{\sum_i S_i + \sum_i ES_i + \sum_i PS_i}$$

Gunawardena (2005) derived conditions to approximate ρ_n by an Hill function of the ratio $\dfrac{E}{P}$(see Section A). Additional sufficient conditions on the variables of the system to approximate σ_n by an Hill function of $\dfrac{E_{tot}}{P_{tot}}$ are thus:

$$\tfrac{E}{P} \simeq \tfrac{E_{tot}}{P_{tot}} \text{ and } \sigma_n \simeq \rho_n$$

In the following, we derive sufficient constraints on the parameter values which satisfy the above constraints on the variables of the system. A summary of these conditions is given in Table S1.

Sufficient conditions for $\frac{E}{P} \simeq \frac{E_{tot}}{P_{tot}}$

Gunawardena derived the following sufficient conditions for $\frac{E}{P} \simeq \frac{E_{tot}}{P_{tot}}$:

$$S_{tot} \ll P_{tot} \text{ and } E_{tot} \leq 2P_{tot}$$

Here we propose a simple derivation of alternative sufficient conditions on the parameters, which do not impose that $S_{tot} \ll P_{tot}$, to obtain the approximation $\frac{E}{P} \simeq \frac{E_{tot}}{P_{tot}}$.

First we have: $E_{tot} = E + \sum_0^{n-1} ES_i$ and $P_{tot} = P + \sum_1^n PS_i$

At steady state, we also have:

$$ES_i = \frac{E \cdot S_i}{K_{Mi}} \quad \text{(S10)}$$
$$PS_i = \frac{P \cdot S_i}{K'_{Mi}} \quad \text{(S11)}$$

Therefore:

$$E_{tot} = E\left(1 + \sum_0^{n-1} \frac{S_i}{K_{Mi}}\right) \quad \text{(S12)}$$
$$P_{tot} = P\left(1 + \sum_1^{n} \frac{S_i}{K'_{Mi}}\right) \quad \text{(S13)}$$

We call:

$$K_{Mmin} = \min_i(K_{Mi}) \tag{S14}$$
$$K'_{Mmax} = \max_i(K'_{Mi}) \tag{S15}$$

Minorating (resp. majorating) K_{Mi} (resp. K'_{Mi}) by K_{Mmin} (resp. K'_{Mmax}), we obtain:

$$E_{tot} \leq E\left(1 + \frac{1}{K_{Mmin}}\sum_0^{n-1} S_i\right) \tag{S16}$$
$$P_{tot} \geq P\left(1 + \frac{1}{K'_{Mmax}}\sum_1^{n} S_i\right) \tag{S17}$$

Majorating $\sum_0^n S_i$ by S_{tot}, we get:

$$E_{tot} \leq E\left(1 + \frac{S_{tot}}{K_{Mmin}}\right) \tag{S18}$$
$$P_{tot} \geq P\left(1 + \frac{S_{tot}}{K'_{Mmax}}\right) \tag{S19}$$

which leads to:

$$\frac{E_{tot}}{P_{tot}} \leq \frac{E}{P}\left(\frac{1 + \frac{S_{tot}}{K_{Mmin}}}{1 + \frac{S_{tot}}{K'_{Mmax}}}\right) \tag{S20}$$

Therefore, a sufficient condition to get $\frac{E}{P} \simeq \frac{E_{tot}}{P_{tot}}$ is:

$$S_{tot} \ll \min(K_{Mmin}, K'_{Mmax}) \tag{S21}$$

For the parameter values chosen to simulate the multisite phosphorylation module, we have $S_{tot} = 1$ and $\min(K_{Mmin}, K'_{Mmax}) = 200$ (see Figure 3).

Sufficient conditions for $\sigma_n \simeq \rho_n$

A sufficient condition on the variables to get $\sigma_n \simeq \rho_n$ is $\sum_i ES_i + \sum_i PS_i \ll \sum_i S_i$.

At steady state, we have:

$$ES_i = \frac{E \cdot S_i}{K_{Mi}} \tag{S22}$$
$$PS_i = \frac{P \cdot S_i}{K'_{Mi}} \tag{S23}$$

which leads to:

$$\sum_0^{n-1} ES_i = E\sum_0^{n-1} \frac{S_i}{K_{Mi}} \tag{S24}$$
$$\sum_1^{n} PS_i = P\sum_1^{n} \frac{S_i}{K'_{Mi}} \tag{S25}$$

Majorating E and P by E_{tot} and P_{tot} respectively, we obtain:

$$\sum_0^{n-1} ES_i \leq E_{tot} \sum_0^{n-1} \frac{S_i}{K_{Mi}} \tag{S26}$$

$$\sum_1^{n} PS_i \leq P_{tot} \sum_1^{n} \frac{S_i}{K'_{Mi}} \tag{S27}$$

We call:

$$K'_{Mmin} = \min_i(K'_{Mi}) \tag{S28}$$

Minorating K_{Mi} and K'_{Mi} by K_{Mmin} and K'_{Mmin} respectively, we get:

$$\sum_0^{n-1} ES_i \leq \frac{E_{tot}}{K_{Mmin}} \sum_0^{n} S_i \tag{S29}$$

$$\sum_1^{n} PS_i \leq \frac{P_{tot}}{K'_{Mmin}} \sum_0^{n} S_i \tag{S30}$$

Summing the previous inequalities, we obtain:

$$\sum_0^{n-1} ES_i + \sum_1^{n} PS_i \leq \left(\frac{E_{tot}}{K_{Mmin}} + \frac{P_{tot}}{K'_{Mmin}} \right) \sum_0^{n} S_i \tag{S31}$$

Thus, sufficient conditions on the parameters to get $\sigma_n \simeq \rho_n$ are:

$$E_{tot} \ll K_{Mmin} \text{ and } P_{tot} \ll K'_{Mmin}$$

For the parameter values chosen to simulate the multisite phosphorylation module, we have $S_{tot} = 1$, $E_{tot} \simeq 1$, $K_{Mmin} = 200$, and $K'_{Mmin} = 20$ for $n = 10$, $K'_{Mmin} = 2$ for $n = 4$ (see Figure 3).

C. Goodwin model and multisite phosphorylation: Quasi-steady state assumption

We show here that the 3-variable Goodwin model is the quasi-steady state limit of the developed model.

We first recall the equations of the Goodwin model combined with the multisite phosphorylation module:

$$\begin{aligned}
\frac{dX}{dt} &= k_1 S_{act} - k_2 X \\
\frac{dY}{dt} &= k_3 X - k_4 Y \\
\frac{dE}{dt} &= k_5 Y + \sum_{i=0}^{n-1} (k_{d,i} \cdot ES_i - k_{a,i} \cdot E \cdot S_i + k_{c,i} \cdot ES_i) - k_6 E \\
\frac{dS_0}{dt} &= k_{d,0} \cdot ES_0 - k_{a,0} \cdot E \cdot S_0 + k'_{c,0} \cdot PS_1 + k_6 ES_0 \\
\frac{dS_i}{dt} &= k_{c,i-1} \cdot ES_{i-1} + k_{d,i} \cdot ES_i - k_{a,i} \cdot E \cdot S_i + k'_{c,i} \cdot PS_{i+1} \\
&\quad + k'_{d,i-1} \cdot PS_i - k'_{a,i-1} \cdot P \cdot S_i + k_6 ES_i \qquad \text{for } i \in \{1, 2, \ldots, n-1\} \\
\frac{dS_n}{dt} &= k'_{d,n-1} \cdot PS_n - k'_{a,n-1} \cdot P \cdot S_n + k_{c,n-1} \cdot ES_{n-1} \\
\frac{dES_i}{dt} &= k_{a,i} \cdot E \cdot S_i - (k_{d,i} + k_{c,i}) \cdot ES_i - k_6 ES_i \qquad \text{for } i \in \{0, 1, \ldots, n-1\} \\
\frac{dPS_i}{dt} &= k'_{a,i-1} \cdot P \cdot S_i - (k'_{d,i-1} + k'_{c,i-1}) \cdot PS_i \qquad \text{for } i \in \{1, 2, \ldots, n\}
\end{aligned} \tag{S32}$$

where

$$S_{act} = \sum_{i=0}^{n-1} S_i \tag{S33}$$

Note that

$$S_{tot} = \sum_{0}^{n} S_i + \sum_{0}^{n-1} ES_i + \sum_{1}^{n} PS_i$$

and

$$P_{tot} = P + \sum_{1}^{n} PS_i$$

are constant.

Assuming that the enzymatic reactions occur at a much faster time-scale than the other processes involved in the model, which is achieved when

$$(k_1, k_2, k_3, k_4, k_5, k_6) \ll (k_{a,i}, k_{d,i}, k_{c,i}, k'_{a,i}, k'_{d,i}, k'_{c,i})$$

the equations reduce to:

$$\begin{aligned}
\frac{dX}{dt} &= k_1 S_{act} - k_2 X \\
\frac{dY}{dt} &= k_3 X - k_4 Y \\
\frac{dE}{dt} &= k_5 Y - k_6 E \\
\frac{dS_0}{dt} &= k_6 ES_0 \\
\frac{dS_i}{dt} &= k_6 ES_i \qquad \text{for } i \in \{1, 2, \dots, n-1\} \\
\frac{dS_n}{dt} &= 0 \\
\frac{dES_i}{dt} &= -k_6 ES_i \qquad \text{for } i \in \{0, 1, \dots, n-1\} \\
\frac{dPS_i}{dt} &= 0 \qquad \text{for } i \in \{1, 2, \dots, n\}
\end{aligned} \tag{S34}$$

for $i \in \{0, 1, \dots, n\}$, where

$$S_{act} = \sum_{i=0}^{n-1} S_i \tag{S35}$$

Following the quasi-steady state approximations and for appropriate parameter setting which satisfies

$$(PS_i, ES_i) \ll S_{tot} \tag{S36}$$

(see Section B), we can make the following approximation:

$$\sum_0^n S_i \approx S_{tot}.$$

We can then rewrite S_{act} as follows:

$$S_{act} = \sum_0^{n-1} S_i = \sum_0^n S_i - S_n \approx S_{tot}\,(1 - \rho_n)$$

with:

$$\rho_n = \frac{S_n}{S_0 + S_1 + S_2 + \dots + S_n} \tag{S37}$$

ρ_n can be approximated by an Hill function of the ratio $\frac{E}{P}$ (see Section A):

$$\rho_n \approx \frac{\left(\frac{E}{P}\right)^n}{1 + \left(\frac{E}{P}\right)^n} \tag{S38}$$

For appropriate parameter setting which satisfies

$$\frac{E}{P} \approx \frac{E_{tot}}{P_{tot}} \tag{S39}$$

(see Section B) and after summing the equations of the different forms of enzyme E ($E_{tot} = E + \sum_0^{n-1} ES_i$), the system becomes:

$$\begin{aligned}
\frac{dX}{dt} &= k_1 S_{tot} \frac{1}{1 + \left(\frac{E_{tot}}{P_{tot}}\right)^n} - k_2 X \\
\frac{dY}{dt} &= k_3 X - k_4 Y \\
\frac{dE_{tot}}{dt} &= k_5 Y - k_6 E_{tot}
\end{aligned} \tag{S40}$$

For $S_{tot} = 1$, $P_{tot} = 1$ and $E_{tot} = Z$, the model is identical to the original 3-variable model. Conditions on parameter values which fulfil conditions (S36) and (S39) can be found in Section B.

D. Goodwin model and multisite phosphorylation: Michaelis-Menten kinetics

In the paper, we have analyzed the fully developed version of the model, in which each reaction step is explicitely described by mass-action laws. In the present section we show that using Michaelis-Menten kinetics for each phosphorylation/dephosphorylation reaction leads to very consistent results.

The Michaelis-Menten-based model counts $n+4$ variables whose dynamics is governed by the following equations:

$$\begin{aligned}
\frac{dX}{dt} &= k_1 S_{act} - k_2 X \\
\frac{dY}{dt} &= k_3 X - k_4 Y \\
\frac{dE}{dt} &= k_5 Y - k_6 E \\
\frac{dS_0}{dt} &= -v_{m,0}\frac{S_0}{K_{M,1}+S_0} + v'_{m,0}\frac{S_1}{K'_{M,1}+S_1} \\
\frac{dS_i}{dt} &= v_{m,i-1}\frac{S_{i-1}}{K_{M,i-1}+S_{i-1}} - v'_{m,i-1}\frac{S_i}{K'_{M,i-1}+S_i} - v_{m,i}\frac{S_i}{K_{M,i}+S_i} + v'_{m,i}\frac{S_{i+1}}{K'_{M,i}+S_{i+1}} \\
&\quad \text{for } \ i \in \{1,2,\ldots,n-1\} \\
\frac{dS_n}{dt} &= v_{m,n-1}\frac{S_{n-1}}{K_{M,n-1}+S_{n-1}} - v'_{m,n-1}\frac{S_n}{K'_{M,n-1}+S_n}
\end{aligned} \tag{S41}$$

where

$$S_{act} = \sum_{i=0}^{n-1} S_i \tag{S42}$$

where the parameters of the developed model and the Michaelis-Menten parameters are linked by the following equations:

$$v_{m,i} = k_{c,i}E \text{ and } v'_{m,i} = k'_{c,i}P$$
$$K_{M,i} = \frac{k_{d,i}+k_{c,i}}{k_{a,i}} \text{ and } K'_{M,i} = \frac{k'_{d,i}+k'_{c,i}}{k'_{a,i}}$$

A comparison of the limit cycles obtained with the fully developed model and with the corresponding Michaelis-Menten variant shows a perfect agreement between the two models (Fig. S2).

E. Zero-order ultrasensitivity in the single site phosphorylation system

Goldbeter and Koshland (1981) showed how ultrasensitivity may arise in a system based on the covalent modification of a protein. They consider a protein that can exist in two forms, e.g. a phosphorylated form (S_1) and a unphosphorylated form (S_0), and that the interconversions are catalyzed by a kinase E and a phosphatase P.

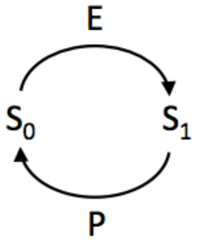

Assuming a Michaelis-Menten molecular mechanism, the detailed reaction scheme is as follows:

$$\begin{aligned} & S_0 + E \underset{k_d}{\overset{k_a}{\rightleftharpoons}} ES_0 \overset{k_c}{\rightarrow} S_1 + E \\ & S_1 + P \underset{k'_d}{\overset{k'_a}{\rightleftharpoons}} PS_1 \overset{k'_c}{\rightarrow} S_0 + P \end{aligned} \tag{S43}$$

The corresponding equations of evolution are:

$$\begin{aligned} \frac{dS_0}{dt} &= -k_a \cdot S_0 \cdot E + k_d \cdot ES_0 + k'_c \cdot PS_1 \\ \frac{dES_0}{dt} &= k_a \cdot S_0 \cdot E - (k_d + k_c) \cdot ES_0 \\ \frac{dS_1}{dt} &= -k'_a \cdot S_1 \cdot P + k'_d \cdot PS_1 + k_c \cdot ES_0 \\ \frac{dPS_1}{dt} &= k'_a \cdot S_1 \cdot P - (k'_d + k'_c) \cdot PS_1 \end{aligned} \tag{S44}$$

The total concentration of the different forms of S, E, and P are constant:

$$\begin{aligned} S_{tot} &= S_0 + S_1 + ES_0 + PS_1 \\ E_{tot} &= E + ES_0 \\ P_{tot} &= P + PS_1 \end{aligned} \tag{S45}$$

At steady state, we have:

$$k_a S_0 \cdot E - k_d ES_0 = k_c ES_0 \tag{S46}$$

$$k'_a S_1 \cdot P - k'_d PS_1 = k'_c PS_1 \tag{S47}$$

$$k_c ES_0 = k'_c PS_1 \tag{S48}$$

Using the conservation equations of E and P, we can eliminate E and P in the above equations to obtain:

$$\begin{aligned} ES_0 &= \frac{S_0 E_{tot}}{K_{m1} + S_0} \\ PS_1 &= \frac{S_1 P_{tot}}{K_{m2} + S_1} \end{aligned} \tag{S49}$$

with

$$K_M = \left(\frac{k_c + k_d}{k_a}\right) \quad \text{and} \quad K'_M = \left(\frac{k'_c + k'_d}{k'_a}\right) \tag{S50}$$

We define the maximum rates of E and P:

$$\begin{aligned} v &= k_c E_{tot} \\ v' &= k'_c P_{tot} \end{aligned} \tag{S51}$$

Using relations (S49), relation (S48) rewrites:

$$v \frac{S_0}{K_M + S_0} \quad = \quad v' \frac{S_1}{K'_M + S_1} \tag{S52}$$

Suppose

$$ES_0, PS_1 << S_0, S_1 \tag{S53}$$

which is acheived when

$$S_{tot} >> E_{tot}, P_{tot} \tag{S54}$$

Then the conservation equation of S can be approximated by:

$$S_{tot} \approx S_0 + S_1 \tag{S55}$$

Taking $S_{tot} = 1$ and eliminating S_0 in Eq. (S52), we obtain

$$\frac{v(1 - S_1)}{K_M + (1 - S_1)} = v' \frac{S_1}{K'_M + S_1} \tag{S56}$$

which rearranges to a second-degree equation of S_1:

$$S_1^2 \left(\frac{v}{v'} - 1\right) - S_1 \left[\left(\frac{v}{v'} - 1\right) - K'_M \left(\frac{v}{v'} + \frac{K_M}{K'_M}\right)\right] - K'_M \left(\frac{v}{v'}\right) \tag{S57}$$

Let's call

$$\phi = \left(\frac{v}{v'} - 1\right) - K'_M \left(\frac{v}{v'} + \frac{K_M}{K'_M}\right) \tag{S58}$$

Then it can be shown that Eq. (S57) admits a unique positive solution:

$$S_1 = \frac{\phi + \left[\phi^2 + 4\left(\frac{v}{v'} - 1\right) K'_M \left(\frac{v}{v'}\right)\right]^{1/2}}{2\left(\frac{v}{v'} - 1\right)} \tag{S59}$$

with

$$S_0 = 1 - S_1 \tag{S60}$$

In the case where $K_M, K'_M << 1$, the curve for S_1 (defined by Eq. (S59)) takes the form of a sigmoid with a very sharp threshold (ultra-sensitivity) (Fig. S3).

The conditions to obtain the ultra-sensitive response curve are recapitulated in Table S1. For $K_M = K'_M$, it can be shown that S_1 admits an inflexion point at $\frac{v}{v'} = 1$ and that the slope of S_1 at this point is: $\frac{1}{8} \cdot \frac{1 + 2K_M}{K_M}$. Therefore S_1 approximates an Hill function of Hill coefficient n if the slopes at the inflexion point of these two functions are closed i.e. if:

$$\frac{1}{8} \cdot \frac{1 + 2K_M}{K_M} \approx \frac{1}{4n} \left(n + 1\right)^{1+\frac{1}{n}} \left(n - 1\right)^{1-\frac{1}{n}} \tag{S61}$$

This condition has been used to calculate an initial guess for K_M ($K_M = 0.056$). The parameter values eventually chosen in the simulation (Fig. 8) is of the same order of magnitude as the initial guess value ($K_M = 0.02$).

F. Bistability in the 2-site phosphorylation system

In the present section, we show how the 2-site phosphorylation system can generate bistability.

We consider the following mechanism in which a kinase E and the phosphatase P catalyze the phosphorylation and dephosphorylation steps respectively.

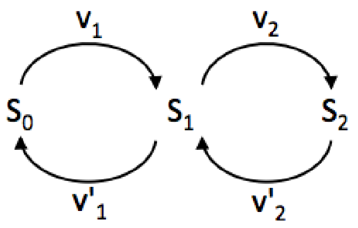

The detailed reaction scheme is:

$$\mathrm{S_0{+}E} \underset{k_{d1}}{\overset{k_{a1}}{\rightleftharpoons}} \mathrm{ES_0} \overset{k_{c1}}{\rightarrow} \mathrm{S_1 + E} \tag{S62}$$

$$\mathrm{S_1{+}E} \underset{k_{d2}}{\overset{k_{a2}}{\rightleftharpoons}} \mathrm{ES_1} \overset{k_{c2}}{\rightarrow} \mathrm{S_2 + E} \tag{S63}$$

$$\mathrm{S_1{+}P} \underset{k'_{d1}}{\overset{k'_{a1}}{\rightleftharpoons}} \mathrm{PS_1} \overset{k'_{c1}}{\rightarrow} \mathrm{S_0 + P} \tag{S64}$$

$$\mathrm{S_2{+}P} \underset{k'_{d2}}{\overset{k'_{a2}}{\rightleftharpoons}} \mathrm{PS_2} \overset{k'_{c2}}{\rightarrow} \mathrm{S_1 + P} \tag{S65}$$

Under the quasi-steady state assumption, the kinetic equations (17) modelling the evolution of this system reduce to (Ortega et al, 2006):

$$\begin{aligned} \frac{dS_0}{dt} &= v'_1 - v_1 \\ \frac{dS_1}{dt} &= v_1 - v'_1 - v_2 + v'_2 \\ \frac{dS_2}{dt} &= v_2 - v'_2 \end{aligned} \tag{S66}$$

with:

$$\begin{aligned} v_1 &= \frac{v_{m1}\dfrac{S_0}{K_{M1}}}{1+\dfrac{S_0}{K_{M1}}+\dfrac{S_1}{K_{M2}}}, \qquad v'_1 = \frac{v'_{m1}\dfrac{S_1}{K'_{M1}}}{1+\dfrac{S_2}{K'_{M1}}+\dfrac{S_1}{K'_{M2}}} \\ v_2 &= \frac{v_{m2}\dfrac{S_1}{K_{M2}}}{1+\dfrac{S_0}{K_{M1}}+\dfrac{S_1}{K_{M2}}}, \qquad v'_2 = \frac{v'_{m2}\dfrac{S_2}{K'_{M2}}}{1+\dfrac{S_2}{K'_{M1}}+\dfrac{S_1}{K'_{M2}}} \end{aligned} \tag{S67}$$

where K_{M1}, K_{M2}, K'_{M1} and K'_{M2} are the Michaelian constants:

$$\begin{aligned} K_{M1} &= \frac{k_{d1}+k_{c1}}{k_{a1}} \;, \qquad K_{M2} = \frac{k_{d2}+k_{c2}}{k_{a2}} \\ K'_{M1} &= \frac{k'_{d1}+k'_{c1}}{k'_{a1}} \;, \qquad K'_{M2} = \frac{k'_{d2}+k'_{c2}}{k'_{a2}} \end{aligned} \tag{S68}$$

and v_{m1}, v_{m2}, v'_{m1} and v'_{m2} are the maximum rates of phosphorylation and dephosphorylation:

$$\begin{aligned} v_{m1} &= k_{c1}E_{tot} \;, \qquad v_{m2} = k_{c2}E_{tot} \\ v'_{m1} &= k'_{c1}P_{tot} \;, \qquad v'_{m2} = k'_{c2}P_{tot} \end{aligned} \tag{S69}$$

The reader can refer to Section A of Ortega et al. (2006) for details on this derivation.

Under additional constraints on parameter values, Ortega et al. (2006) derived conditions for the system to display bistability. Introducing an asymmetric factor θ defined as:

$$\theta = \frac{v_{m2}v'_{m1}}{v_{m1}v'_{m2}} \tag{S70}$$

and under the following assumptions:

$$\begin{aligned} &E_{tot}, P_{tot} \ll S_{tot} \\ &K_{M1} = K_{M2} = K'_{M1} = K'_{M2} = K_S \end{aligned} \tag{S71}$$

Ortega et al. determined the following necessary and sufficient conditions on θ and K_S to obtain a region of $\frac{v_{m1}}{v'_{m2}} = \frac{k_{c1}E_{tot}}{k'_{c2}P_{tot}}$ values where the system display bistabillity:

$$\begin{aligned} \theta &> \frac{(1+K_S)^2}{(1-2K_S)^2} \\ K_S &> \frac{1}{2} \end{aligned} \tag{S72}$$

Thus there exists a region of E_{tot} values in which two stable steady states coexist iff the above conditions are fulfilled. These conditions are summarized in Table S1. The parameter values chosen for the simulation of the bistable module fulfil these conditions (Figures 12, 13 and 15).
